# Translational research approaches to study pediatric polycystic kidney disease

**DOI:** 10.1186/s40348-021-00131-x

**Published:** 2021-12-09

**Authors:** Max Christoph Liebau, Djalila Mekahli

**Affiliations:** 1 Department of Pediatrics, Center for Rare Diseases and Center for Molecular Medicine, University Hospital Cologne and Medical Faculty, University of Cologne, Kerpener Str. 62, 50937 Cologne, Germany; 2 Department of Pediatric Nephrology and Organ Transplantation, University Hospitals Leuven, Herestraat 49, 3000 Leuven, Belgium; 3 Department of Development and Regeneration, PKD Research Group, Laboratory of Pediatrics, KU Leuven, Leuven, Belgium

**Keywords:** PKHD1, PKD1, PKD2, Ciliopathies, Genetic Kidney Disease, ARegPKD, ADPedKD

## Abstract

Polycystic kidney diseases (PKD) are severe forms of genetic kidney disorders. The two main types of PKD are autosomal recessive and autosomal dominant PKD (ARPKD, ADPKD). While ARPKD typically is a disorder of early childhood, patients with ADPKD often remain pauci-symptomatic until adulthood even though formation of cysts in the kidney already begins in children. There is clinical and genetic overlap between both entities with very variable clinical courses. Subgroups of very early onset ADPKD may for example clinically resemble ARPKD. The basis of the clinical variability in both forms of PKD is not well understood and there are also limited prediction markers for disease progression for daily clinical life or surrogate endpoints for clinical trials in ARPKD or early ADPKD.

As targeted therapeutic approaches to slow disease progression in PKD are emerging, it is becoming more important to reliably identify patients at risk for rapid progression as they might benefit from early therapy. Over the past years regional, national and international data collections to jointly analyze the clinical courses of PKD patients have been set up. The clinical observations are complemented by genetic studies and biorepositories as well as basic science approaches to elucidate the underlying molecular mechanisms in the PKD field. These approaches may serve as a basis for the development of novel therapeutic interventions in specific subgroups of patients. In this article we summarize some of the recent developments in the field with a focus on kidney involvement in PKD during childhood and adolescence and findings obtained in pediatric cohorts.

## Introduction

Polycystic kidney diseases (PKD) are severe systemic disorders that predominantly affect the kidneys and the liver [1]. The two main forms of PKD are autosomal recessive PKD (ARPKD; OPRHA:731) and autosomal dominant PKD (ADPKD, OPRHA:730).

ARPKD is usually diagnosed prenatally or in the first year of life. The disease is characterized by bilateral fibrocystic changes of the kidneys typically presenting with massive organ enlargement due to ubiquitous microcysts mainly developing from the collecting duct. Impairment of kidney function is variable. Antenatal decline of kidney function is associated with oligo-/anhydramnios which subsequently results in pulmonary hypoplasia. Early pulmonary disease in ARPKD is still associated with substantial mortality even in very advanced medical centers. In addition, hepatic involvement due to a developmental defect of bile ducts is an obligatory finding in ARPKD, which may clinically result in hepatic fibrosis with portal hypertension and bile duct dilatations with an increased risk of cholangitis [1–3].

ADPKD is a more slowly developing disease with clinical symptoms typically becoming prominent in adulthood, even though cyst development starts in childhood or even *in utero* [1, 4]. The disease is characterized by progressive development of macrocysts in the kidney developing from all parts of the tubule. Kidney volume increases with cyst volume. Fibrotic changes in the kidney develop during the course of the disease. Extrarenal manifestations include - amongst others - the development of hepatic cysts, diverticula and hernia, cardiovascular anomalies, and pain (Table 1) [1, 5].

**Table 1:** Comparison of typical clinical features of ARPKD and ADPKD

	ARPKD OPRHA:731	ADPKD OPRHA:730
*Incidence*	1:20.000	1:500-1:1000
Main clinical kidney manifestations	Prenatal enlarged kidneys, cystic kidneys, oligo-/anhydramnios Chronic kidney disease Hyponatremia Hypertension	Increased TKV, cystic kidneys Hypertension Proteinuria Hematuria Chronic kidney disease
Kidney Ultrasound	Increased echogenicity of kidney parenchyma. „Salt-and-pepper“-pattern. Small, sometimes invisible cysts (<2mm). More ADPKD-like pattern with advancing age	Cysts of different sizes in cortex and medulla. Usually several large cysts. Usually bilateral cysts
Hepatic Pathology	Mandatory: Ductal plate malformation/congenital hepatic fibrosis with hyperplastic biliary ducts and portal fibrosis Dilated bile ducts (Caroli syndrome) Portal hypertension Increased risk of cholangitis	Occasionally ductal plate malformation/congenital hepatic fibrosis Liver cysts: Common in adults, rare in children.
Associated anomalies	Neonatal respiratory distress/failure due to pulmonary hypoplasia Rarely pancreatic cysts. Single case reports of intracranial aneurysms.	Pancreatic cysts and/or cysts in other epithelial organs Colon diverticula and hernia Cardiovascular anomalies, and familiarly clustered intracranial aneurysms, abdominal Aorta aneurysms Bronchiectasis Pain

The genes mainly affected in ARPKD and ADPKD are well-known (*PKHD1* (ARPKD), *PKD1* and *PKD2* (ADPKD)), but the pronounced and poorly understood clinical variability cannot be fully explained by underlying genotypes. Variants in additional genes and first modifier genes have been identified [1]. Overlapping phenotypes between ARPKD and rapidly progressing ADPKD, and patients with coincidental variants in multiple PKD genes that show aggravated phenotypes have been described [6]. Biallelic hypomorphic *PKD1* variants have been identified as a common cause of early-onset PKD [7]. In addition to genetic aspects, unknown environmental factors may influence the phenotype.

In this manuscript we focus on kidney involvement in the two main forms of PKD. ARPKD and ADPKD are major contributors to chronic kidney disease (CKD) with ARPKD being an important cause of CKD specially in very young children and ADPKD being the by far most common genetic cause for chronic kidney failure (CKF) in adults. Despite important work by multiple groups [8–11], there has been a knowledge gap on longitudinal courses of ARPKD and pediatric ADPKD and prognostic markers. Clinically, there is limited published data on severe and rapidly-progressing early-onset ADPKD. For typical ADPKD that progresses to kidney failure at a mean age of 58.1 years for *PKD1* patients and at 79.9 years for *PKD2* patients first risk scores have been developed in adult patients [12, 13]. Yet, these scores cannot be fully applied in pediatric and young adult patients as they include age-dependent variables. Height-adjusted total kidney volume (HtTKV) has been associated with hypertension of ADPKD already in children [14, 15], but data on in-depth longitudinal clinical characterization of children suffering from ADPKD remains sparse.

There is a clear need for evidence-based and targeted treatment in both forms of PKD during childhood and adolescence. ARPKD is a very severe disorder of early childhood. In ADPKD on the other hand cystogenesis starts early in life and kidney function mainly declines once structural changes in the kidney parenchyma are pronounced. It has therefore been suggested that young ADPKD patients could highly profit from early interventions that would retard the development of structural changes in the kidney with subsequent positive long-term effects.

Clinical research on ARPKD and pediatric ADPKD has for a long time been hampered by the lack of well-defined primary end points for trials. Most of the current treatment approaches for pediatric PKD therefore remain symptomatic and opinion-based. The current treatment of severely affected children with ADPKD is based on strict antihypertensive therapy [4, 16].

For treatment of adult ADPKD patients multiple clinical trials are ongoing. The vasopressin V2-receptor antagonist Tolvaptan has been shown to retard cyst growth and the loss of kidney function in large cohorts of adult ADPKD patients [1]. A clinical trial on the use of Tolvaptan in children with ADPKD is currently ongoing (EudraCT 2016-000187-42). While tolvaptan has shown a clinical benefit for adult patients with ADPKD, it is associated with relevant side effects including polydipsia and polyuria. Adult patients produce 4-8 liters of urine a day and rare but severe hepatic side effects have been described [1, 16].

Data from preclinical studies show that common molecular and cellular mechanisms in ARPKD and ADPKD contribute to the pathogenesis of the different subtypes of PKD [1, 17]. Thus, a transfer of knowledge from ADPKD to ARPKD seems plausible. However, given the current difficulties to predict disease courses and to identify children at special risk of very rapid disease progression in both forms of PKD, novel targeted therapies could either be withheld or expose individuals to potential side effects without providing any benefit. A deeper understanding of the natural disease history and rapid, accurate and prognostic diagnostic measures are therefore urgently required to guide counseling, the timing of diagnosis and monitoring, and to work towards personalized therapeutic approaches. A combination of longitudinal clinical phenotyping and the survey of international cohorts together with a translational research approach to identify underlying molecular disease mechanisms and biomarkers appears ideal to identify diagnostic criteria that can be applied early in life.

Over the last years we have established the international registry studies ARegPKD (www.ARegPKD.org) and ADPedKD (www.ADPedKD.org) [18, 19]. In brief, we aim to follow patients with the clinical diagnosis of ARPKD or pediatric ADPKD to describe the longitudinal clinical courses. By End of May 2021 more than 680 patients have been included in ARegPKD and more than 1200 patients have been registered for the more recently launched global ADPedKD network.

## Recent insights obtained from pediatric PKD cohort studies

### ARPKD

As ARegPKD has already been running for a few years, the first publications have emerged and can demonstrate the strength and the potential of the chosen international approach. Clinical research in the ARPKD field has been facing the typical problems associated with rare and variable disorders. ARPKD is a very rare disease with an estimated incidence of 1:20.000 [1]. Despite very important work by various groups [8–10] it has been a challenge to gather detailed longitudinal clinical data on large cohorts. This has impeded the definition of primary endpoints for clinical trials. A very clearly-defined hard primary endpoint is needed for a clinical trial. To allow studying in a cohort in a clinical trial such an end point would need to be reached by a substantial percentage of a study cohort in a reasonable period of time. As numbers in a potential trial on a rare disease like ARPKD will remain limited it appears even more important to clearly define comparable subcohorts that would be at a high risk to reach such an endpoint. Thus, in a first step it is crucial to describe the clinical course of the disease under standard of care conditions and to identify potential clinical, genetic or biochemical risk factors for severe disease progression, which may either affect the kidneys, the liver or both organs. ARegPKD analyses have described the ARPKD phenotype in adults and studied the potential consequences of very early bilateral nephrectomy in ARPKD patients [20, 21]. To identify potential risk markers, genetics is an obvious candidate for a recessive disorder. Genotype-phenotype correlations have for a long time been limited to the type of variant in ARPKD with biallelic *PKHD1* variants being associated with severe phenotypes [1]. Indeed, surviving ARPKD patients with biallelic *PKHD1* variants have only recently been described [22, 23]. ARegPKD data has recently highlighted the importance of the localization of the variant in addition to its type in patients with missense variants [24]. To study potential genotype-phenotype associations, the gene was categorized into four sections. Patients with either two missense variants in one section or a missense variant and a null variant were analyzed together under the assumption that the weaker variant would define the phenotype in a recessive disorder. The data show that patients with variants affecting the amino acids 709-1837 of the ARPKD protein fibrocystin showed better renal outcome, whereas patients with variants affecting amino acids 2625-4074 showed poorer hepatic outcome. The data may explain some of the variability between the kidney and the liver phenotypes that has been described for ARPKD. The findings may also be a helpful contributor to a risk stratification for ARPKD patients and will open various research fields to study the cellular protein function of fibrocystin, e.g. in preclinical mouse models.

In addition to genetics easy-to-obtain clinical data are highly important for stratification of patients into risk groups. A recent study on 385 ARegPKD patients identified prenatal sonographic identification of enlarged kidneys, kidney cysts as well as documentation of oligo-/anhydramnios as candidates for prenatal markers to predict early dialysis dependency in ARPKD patients [25]. Thirty-six patients with the need for dialysis in the first year of life were compared to 349 patients that did not require dialysis in this time frame. Various markers showed an increased hazard ratio for early dialysis dependency in a multivariate Cox regression analysis with documentation of oligohydramnios or anhydramnios, and the need for postnatal respiratory support being most-powerful markers. Interestingly, a predictive model derived from the dataset could identify a gradual increase of probability of early postnatal dialysis dependency according to the isolated or combined documentation of antenatal detection of enlarged kidneys, kidney cysts and documentation of oligo-/anhydramnios [25]. Thus, the clinical data define a first high-risk profile for severe kidney disease in ARPKD and have served as a basis for the establishment of two first clinical phase 3 trials that are aiming to study the safety of Tolvaptan and its effects on the need of kidney replacement therapy in ARPKD patients (NCT04782258, NCT04786574).

Thus, clinical and genetic markers have been identified in ARegPKD to categorize kidney disease progression in ARPKD. Such markers could in the future be supported and reinforced by radiological findings or biochemical markers. Most recently, we identified early childhood htTKV as a potential risk marker for kidney survival in ARPKD [26]. Yet, our understanding of the ARPKD pathophysiology and of the functional role of the ARPKD protein fibrocystin remains limited, hampering the development of novel therapeutic concepts for ARPKD. Work in mouse models that would complement clinical research has for a long time been hampered by the fact that orthologous ARPKD mouse models did not recapitulate the kidney phenotype of ARPKD. More recently, two novel paths have opened up for cellular ARPKD research. The ability to obtain, culture and reprogram epithelial cells from patients’ urine may become an important tool especially for recessive disorders like ARPKD. Furthermore, a novel digenic mouse model that was obtained by crossing an known orthologous ARPKD mouse model with a well-established orthologous ADPKD model shows a kidney phenotype that resembles human ARPKD [27]. This model may become helpful for functional preclinical studies or screening of potential therapeutic approaches. The data again point to a potential overlap between ARPKD and ADPKD in a spectrum of diseases.

### ADPKD

While ADPKD typically becomes clinically symptomatic in adulthood, cystogenesis starts in childhood or even antenatally. Major clinical variability has been described in ADPKD that can only partially be explained by the underlying genetics. It was for a long time believed that children of patients with ADPKD should not be examined but a “wind of change” has recently been noted in this field [28]. More attention has been given to a concept of prevention of disease progression by early modification of ADPKD risk factors. This includes initiating the treatment of modifiable risk factors already in children. The challenges for treating ADPKD in childhood and adolescence have recently been summarized and first specific recommendations on the diagnosis and management of ADPKD in children and young people have been published [4, 16]. Increased htTKV, hypertension and proteinuria are widely-accepted risk markers in adults with ADPKD with more recent emerging comparable data for children with ADPKD. Ambulatory blood pressure measurements and detection of proteinuria in childhood are highly valid examinations and there is a well-established link between hypertension and proteinuria as a consequence of pediatric ADPKD. Furthermore, hypertension and proteinuria are known pediatric risk markers for the progression of CKD. Thus, hypertension and proteinuria may also become promising candidates for surrogate endpoints in clinical trials bridging early disease progression to findings in adults. While it is generally accepted that strict antihypertensive therapy is required and helpful already in pediatric ADPKD in specific, there is a lack of knowledge on the overall clinical development of ADPKD throughout childhood and adolescence. As previously described for ARPKD this knowledge gap is a major challenge to identify and study risk patterns and novel markers in pediatric ADPKD. For adult ADPKD additional urinary, plasma or radiological markers have been described and risk scores like the PROPKD score or the Mayo-TKV-Score are well-established and some of these markers may become interesting candidates for evaluation in childhood and adolescence [1]. Very briefly summarized the PROPKD score is based on four variables: gender, type of genetic variant (truncating *PKD1* variant, non-truncating *PKD1* variant, *PKD2* variant), hypertension before the age of 35 years, first urologic event before the age of 35 years. These variables are weighed differently resulting in a final point score between 0 and 9 points that subsequently allows the classification of patients in three risk categories (low risk, intermediate risk, high risk). A score of ≤3 has a negative predictive value of 81.4% for CKF before 60 years of age, whereas a score >6 shows a positive predictive value of 90.9% for chronic kidney failure before 60 years of age. The Mayo-TKV-Score classifies patients into typical and atypical radiological presentation, with five age-adjusted htTKV subclasses in the typical group (1A-1E in increasing order). Patients in higher classes show more rapid decline of kidney function and thus have a higher risk to develop CKF after 10 years.

For progress in pediatric ADPKD research we will need the association of biobanking and deeply phenotyped patients. The samples obtained during a clinical trial on the efficacy of pravastatin in childhood were for example recently used for metabolic profiling of children and young adults with ADPKD [29]. An additional recent example in the pediatric field includes the evaluation of plasma copeptin, urinary epidermal growth factor (EGF) and urinary MCP-1 as potential early markers in a cross-sectional study of 53 genotyped ADPKD patients with a mean age of 10.4 years. As expected, kidney function was very good in this cohort with a mean eGFR of 122.7 ml/min/1.73m^2^. Patient samples were compared to samples from age-, sex- and BMI-matched healthy controls. While plasma copeptin and urinary EGF did not show major differences, urinary MCP-1 was significantly higher in ADPKD patients compared to controls. This finding was driven by patients with *PKD1* variants independent of their underlying genotype. A group of patients with very early onset ADPKD or early symptomatic ADPKD showed higher urinary MCP-1 levels than asymptomatic patients. Human fetal ADPKD kidneys displayed prominent MCP-1 staining and M2 macrophage infiltration and cellular models with *PKD1* haploinsufficiency exhibited increased MCP-1 secretion. Thus, urinary MCP-1 may become an easily-obtainable marker of disease severity for subgroups of pediatric ADPKD patients [30]. It may in the future be complemented by radiological findings obtained by both novel MR techniques or 3D-ultrasound [28, 30].

## Outlook and Summary

**Figure 1:**
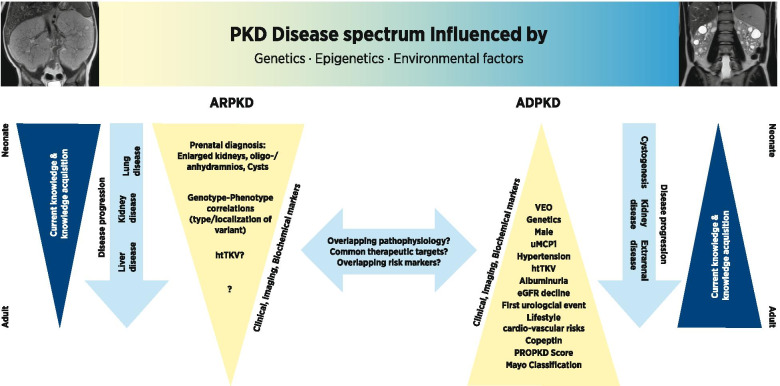
Age-dependent changes of the clinical phenotype (light blue), risk factors for rapid disease progression (yellow) and progression of understanding (dark blue) in ARPKD and ADPKD. Overall ARPKD and ADPKD can be seen as two ends of a disease spectrum with overlapping genetic and clinical features

Over the past ten years there have been major developments in pediatric PKD research. The field has greatly benefitted from the knowledge and the experiences obtained in the general PKD field and in pediatric nephrology. This includes a better understanding of pathomechanisms as well as the identification of prognostic markers of disease progression in adult ADPKD patients. A concept is emerging in which ARPKD and ADPKD may be seen as two ends of a disease spectrum with overlapping genetic and clinical features (Figure 1). The infrastructures generated during the past few years will allow to gain more in-depth insights into the clinical courses of both ARPKD and ADPKD and it appears very likely that clinical risk stratification will soon be possible based on the data obtained in the observational studies. These data will be supported and complemented by the ongoing cell biological work as well as the power of modern multi-omics approaches to identify early prognostic and predictive biomarkers. For ARPKD the goal will be to achieve promising settings for realizable clinical trials with the two trials mentioned above being a first major step ahead. For pediatric ADPKD it will be crucial to keep the balance between safety, and tolerability as well as interference in the daily life of children on the one hand and the benefits of early management on the other hand. Here, establishment of easily-obtainable predictive markers of disease progression in childhood and adolescence as valid primary end points for pediatric clinical trials are urgently needed as a base for the establishment of early targeted treatment. Furthermore, the in-depth clinical characterization of pediatric patients may generate questions for translational research in PKD protein function, thus serving as a stimulus for bidirectional translational research between bench and bedside.

## Data Availability

Data and material serving as the base for this mini review is available by the authors upon reasonable request.

## Abbreviations

ADPKD: Autosomal dominant polycystic kidney disease
ARPKD: Autosomal recessive polycystic kidney disease
CKF: Chronic kidney failure
EGF: Epidermal growth factor
HtTKV: Height-adjusted total kidney volume
PKD: Polycystic kidney disease
PKD1: Polycystic kidney disease 1
PKD2: Polycystic kidney disease 2
PKHD1: Polycystic kidney and hepatic disease 1
